# Superalloy—Steel Joint in Microstructural and Mechanical Characterisation for Manufacturing Rotor Components

**DOI:** 10.3390/ma16072862

**Published:** 2023-04-04

**Authors:** Bożena Szczucka-Lasota, Tadeusz Szymczak, Tomasz Węgrzyn, Wojciech Tarasiuk

**Affiliations:** 1 Faculty of Transport and Aviation Engineering, Silesian University of Technology, Krasińskiego 8, 40-019 Katowice, Poland; 2 Department of Vehicle Type-Approval & Testing, Motor Transport Institute, Jagiellońska 80, 03-301 Warsaw, Poland; 3 Faculty of Mechanical Engineering, Bialystok University of Technology, Wiejska 45c, 15-352 Białystok, Poland

**Keywords:** welding technology, superalloy steel joint, microstructure and mechanical properties, rotor manufacturing

## Abstract

The structure of energy rotor components includes different structural materials in the sections, which are subjected to varying levels of thermal loading. The first component section has to include a precipitation-hardened nickel-based alloy, while the second one may be manufactured from other materials. Due to the installation cost, the use of expensive nickel-based materials is not recommended for applications in sections with a lower degree of thermal loading. Therefore, this aspect is still actually from an engineering point of view and is discussed in the paper by means of manufacturing and experimental approaches. The paper follows the welding problems related to a hybrid joint made of superalloy (Alloy 59) and hard rusting steel (S355J2W+N steel). The problem is solved using the MIG process at various parameters. With respect to the joint quality, microstructural features and mechanical parameters of the examined zone are presented. In the case of microstructure analysis, the dendritic and cellular natures of austenite were dominant elements of the joint. Mechanical tests have expressed a 50% reduction in elongation of the steel and alloy steel weld and lowering mechanical parameters. Mechanical parameters of the joint were on the level of their values observed for the steel, while the hardening coefficient followed the hardening curve of the alloy. Decohesion of the steel and mixed weld has reflected the constant proportion of values of axial and shear stress components up to the total separation. It is noted the tensile curves of the alloy and alloy steel joint follow a very similar shape, reporting the same response on the monotonic tension. The materials can be analysed by applying constitutive equations at very similar values of their coefficients. The obtained results enabled elaborating and examining the MIG welding process for thick-walled structures (not smaller than 8 mm) in detail giving all parameters required for technology. Finally, the technology for producing a hybrid joint using difficult-to-weld materials with different physical and mechanical properties, such as nickel alloys and low-alloy steels, is proposed. Results have shown it possible to develop a technology for producing of hybrid joints (supper alloy + hard rusting steel) with assumed physical and mechanical properties for rotors applied in the power boiler. This solution was proposed instead of previously used elements of rotors from expensive materials. It was assumed that the newly proposed and utilised method of welding will allow for obtaining good properties in terms of energy devices.

## 1. Introduction

Forged rotors, pipes and cast casings used in structures of high-temperature steam power plants as well as in elements of gas turbines or other rotating machines with operating temperatures >700 °C, should be made of nickel-based alloys with the required mechanical parameter and resistance on creep [1,2,3]. At temperatures exceeding the application range of high-temperature-resistant steels, nickel-based alloys are recommended to be used. If the operating values of temperature are higher than 750 °C, nickel-based alloys are required [4] because of their stable precipitates [5,6]. Dispersion-curable alloys have desirable properties for applications above 700 °C, but some disadvantages can also be seen:

- The lack of appropriate production equipment concerning a crack occurrence during the production process [7];
- The wide range of solidification of these alloy types leads to cracks [8];
- Welding of fully cured material promotes the formation of cracks as an effect of the relative inability of the material to compensate for the differential expansion [9].

For significant components such as impellers and housings, which are exposed to high and very high values of temperature, there are often regions with the most elevated and medium operating conditions [10]. In these cases, it is proposed to install elements in multiple subsections, where each area consists of materials with different mechanical, physical and microstructural properties. This structure creates welding problems [5,11].

Rotary air heaters (called rotors) are indispensable elements in the technological system of power boilers. They improve the functioning of the power installation because each degree of heat removed from the flue gas brings measurable economic benefits. The use of an air heater increase, the boiler efficiency by 1% for each 15–25 °C air temperature [5,12].

The durability of rotor welded joints determines the level of boiler efficiency. Structural elements require a welded connection between the section of thermally loaded components. They include a plurality of component sections which, during operation, are exposed to different temperature levels, a first component region being designed for temperatures of >750 °C, and a second component section being designed for temperatures of approximately 600 °C [6]. The first component section includes a precipitation-hardened nickel-based alloy (e.g., Alloy C-276 and Alloy 625 (2.4856) [13,14,15], and the second one represents a low-alloy steel S355J2W+N [16,17,18]. These elements are responsible for the increase in mechanical resistance on high-temperature, lowering cracks occurrence and good resistance to high-temperature corrosion [19].

Unfortunately, during the welding process, the crystallizing metal of the mixed welded joint becomes affected by low values of ultimate tensile stress, which are the result of weld-free shrinkage and cooling of not uniform heated base materials. It leads to cracks and final fractures [20,21].

**Figure 1:**
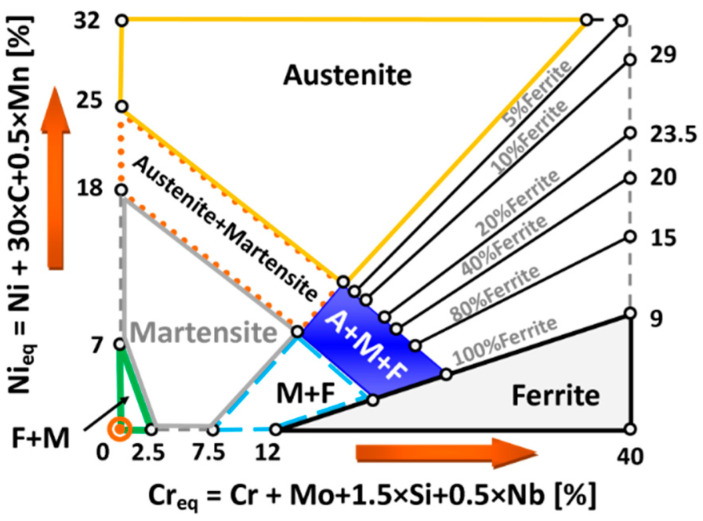
Schaeffler diagram with marked regions for components, A—Austenite, M—Martensite, F—Ferrite (based on [22]).

**Figure 2:**
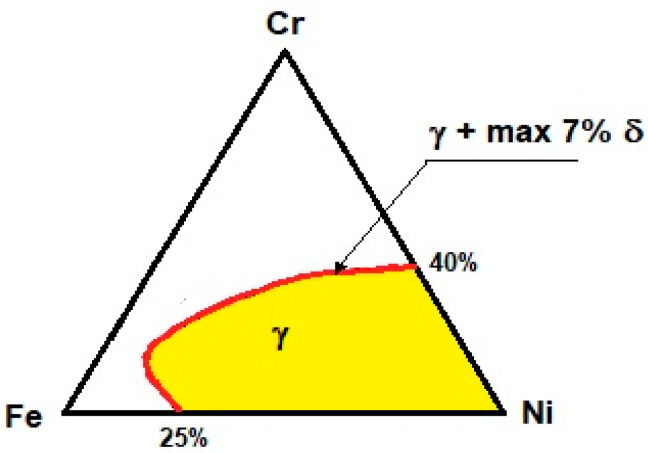
Equilibrium diagram Ni-Fe-Cr (based on [23]).

The observation is in line with Schaeffler’s graph, presented in [22], when welding nickel alloys with steels, an analysis of the Ni-Fe equilibrium graph is insufficient. According to the graph the verification of other elements, especially ferrite-forming ones: chromium and molybdenum are possible. Assuming that molybdenum is the equivalent of chromium ([22], Figure 1), the Fe-Ni-Cr ternary equilibrium system can be analysed (Figure 2).

There is no developed simple method of joining various materials of complex nickel alloys with other steels [10,11,24,25,26].

It can be found in patent JP4206216B2 [1]; the solution makes it possible to join steel with a nickel-based alloy only under the condition of producing the so-called interlayers. This approach can only be applied to flow machines in the power industry.

The hybrid joint of high-alloy, heat-resistant martensitic–ferritic steels and nickel-based superalloys requires several preparatory steps [27]. The joined parts, e.g., IN706 with high-alloy martensitic–ferritic steel St13TNiEL, are first coated by SG-NiCr20Nb surfacing in the area of material joining, and then the material (clad/surfaced) is subjected to high-quality heat treatment (stabilizing annealing at 820 ± 15 °C, cooling to RT, precipitation hardening at 730 ± 15 °C, cooling to RT). During welding, the root layers are applied using the TIG method, and the reinforcement layers are applied using submerged arc welding.

The excellent weldability of the superalloy is a result of its microstructure. According to the diffraction pattern, the microstructure of Inconel 718 contains a dispersion of the γ′ and the γ″ of precipitates in the γ matrix [28,29,30]. Nickel forms solid solutions with copper and saturated solid solutions with the most important alloying elements, including chromium, molybdenum, iron and copper [31,32]. Of these elements, chromium dissolves best in the nickel A1 network (up to 29%). When the concentration of chromium is higher, the α phase that mainly contains chromium, which does not dissolve nickel, becomes present [33,34]. The welding elements from the same material (especially Monel and Inconel) are relatively well-weldable by the most popular arc processes, i.e., MIG, TIG at coated electrodes, covered arc and laser welding [26,35].

The highest tendency to crack is those welds in which cell microstructure is formed during clotting [36,37,38,39]. The cracking of these welds is favoured by relatively smooth surfaces of grain boundaries, with strong segregation of low-melting components. Authors K. Rajasekhar, C. S. Harendranath and R. Raman indicate that to avoid cracking in the joint, cell-dendritic microstructure should be formed during the solidification of the weld [38].

The authors: Yang Li Shuangming et al. demonstrated the concentration of low-melting phases per unit area decreases, reducing the tendency to crack [37]. The data presented in [40,41,42] exhibits that the joint including 40% of nickel content has a tendency to crack.

The last assumption implies another problem in developing a technology for joining the two materials because of enormously different values of thermal coefficients. The average coefficient of Alloy 59 thermal expansion is about 13.1 × 10^−6^ 1/K, whereas for hard-rusting steels, it is around 10.3 × 10^−6^ 1/K. As a result, in thick-walled joints, a risk of cracks appearing during the solidification of the weld (crystallisation cracks) or during reheating of base material and weld (segregation cracks) occurs.

The joining of dissimilar materials is challenging to attain quality joints. There are a lot of joining methods applied for the fabrication of dissimilar joints of different materials [26,28,29,30,31,32,33]. The latest research presented in the world literature (Table 1) concerns obtaining Inconel superalloy grades connections between [34,35,36,37,38,39,40,41,42,43,44,45,46,47,48,49,50,51,52]:

- Different Inconel superalloy grades;
- Inconel alloys with various austenitic sheets of steel.

These joints are obtained using different welding processes (TIG, laser welding, microwave, electrically assisted pressure joining(EAPJ), friction stir welding (FSW) and so on [53,54,55,56,57]. The processes, their parameters and modifications are very well described in the literature. Still, the research gap is the description of simple welding processes used to join low-alloy steel materials with Inconel [58,59,60,61].

**Table 1:** Methods, materials and final welding joint concerning superalloy and alloy steel at rotor application.

Authors	Method	Materials Used and Superalloy Steel	Rotor Application
Handa V., et al. [53]	Microwave hybrid heating	Inconel with another Inconel or with austenite steel No	No
Li Y.-F., et al. [54]	Electrically assisted solid-state joining or electrically assisted pressure joining (EAPJ)	316L steel and Inconel 718 No	No
Müller R., et al. [55]	Multilayer electron beam cladding (EBC)	Inconel 718 with austenitic stainless steel No	No
Kumar N, et al. [53]	Rotary friction welded	Inconel 600 with 316L steel No	The joint is not recommended
Wen Y., et al. [47]	Laser powder with bed fusion materials	Inconel 718 with 316L steel No	No
Raj S., et al. [48]	Welded dissimilar butt joints friction stir welding	Inconel 718 and AISI 204Cu steel No	The joint is not recommended
Anuradha M., et al. [49]	Method TIG	Inconel 718 with high-strength steel No	The joint is not recommended
JP4206216B2 [10]	New method of welding with the interlayers	Inconel with austenitic steel Yes	No
[27,36,37,38]	Method requires several preparatory steps	High-alloy, heat-resistant martensitic–ferritic steels and nickel-based superalloys Yes	No
Tomota Y., et al. [58]	No information	Inconel and Low Alloy Steel Yes	Yes but no details on method
Zhu M. L., et al. [62]	TIG welding and submerged arc welding (SAW) techniques	23CrMoNiWV88 steel and 26NiCrMoV145 Yes	Yes, but only for the examined materials
Nivas R., et al. [61]	GTAW with stress relief annealing or	Inconel 82 or with low alloy steel Yes	No
	SMAW with stress relief annealing	Inconel 182 with low alloy steel Yes	No

Recently, essential articles have appeared on modern structural materials and technological solutions. The problems that may help solve the issue related to DWJ (dissimilar welded joint) welding of unalloyed steel with nickel alloy were dealt with. The authors are focused on the metallurgy of nickel alloy welding, the austenite–ferrite delta microstructure and the thermodynamic conditions of welding. An example of an essential position on the weldability of nickel alloys is the study [63].

It is important to approach the welding of two-phase materials dominated by austenite and δ-ferrites distributed at austenite grain boundaries. The authors of [64] point out that during stainless steel laser arc hybrid welding, welding cracks may occur due to the presence of δ-ferrite. The same phases play a significant role in DWJ welding non-alloy steel with nickel alloy.

In turn, the authors of [62] investigated the behaviour of bimetallic joints CP-Ti/Q235 bimetallic sheets.

Precisely changed various process parameters will allow for the production of a joint of the best quality and properties.

The analysis of the literature and published patents in the field of joining difficult-to-weld nickel alloys with steels clearly shows the lack of guidelines for the welding process and the use of relatively simple methods to obtain correct welds, which can be considered a research gap.

Therefore, this article aims to propose the welding process (MIG) for hybrid joints represented by superalloy and rust resistance low alloy steel. It was assumed that the newly proposed and used method of welding will allow for obtaining good properties in terms of energy devices.

The TIG process is well recognised for the considered problem, but in the article, the authors focused on the MIG welding process. The MIG process is more efficient. The authors presented the role of varying electrode wires and shielding gas mixtures. They have attempted to prove that the MIG method is correct for welding dissimilar joints based on the steel–nickel alloy. Serious attention is paid to the method of bevelling both sheets due to a different heat transfer coefficient, much higher for nickel alloys (90 W/(m·K).) than for low-alloy steel (60 W/(m·K) [65,66].

## 2. Materials, Technology and Methods

Manufacturing the correct DWJ (dissimilar welded joint) is very often a big problem, due to the different materials’ microstructure and their mechanical and physical properties, such as density and thermal conductivity. In the case of the DWJ low-alloy steel and nickel alloy joint, it is important to consider into account the significant difference in heat conduction (1.5 greater for nickel alloys) between the two materials, which may result in high welding stresses, which may provoke cracks. So, as a part of this article, the investigation schema including metallurgical and technological analyses was realised.

**Figure 3:**
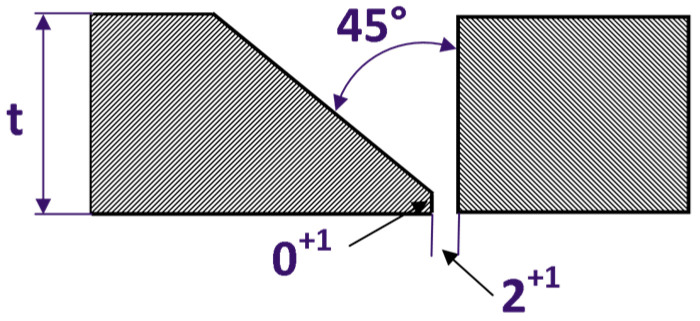
Groove shape.

**Figure 4:**
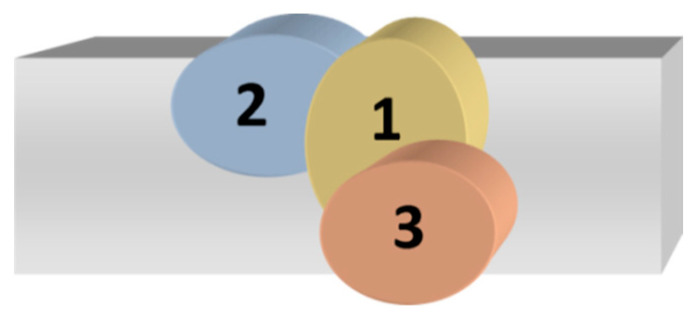
Welding order 3—lower stitch, 1, 2—upper stitch.

In the metallurgical approach, various wires and shielding gases were selected, and as part of the technological approach, the focus was on the role of bevelling and basic welding parameters: arc voltage, current intensity and welding speed. The welding scheme is shown in Figure 3 and Figure 4.

The tested object for the study was a mixed welded joint made by the MIG (131) method, Figure 3 and Figure 4. The joints were made from an 8 mm thick metal sheet and manufactured through the two electrode wires, i.e., NiCr23Mo16 (yield stress 450 MPa, ultimate tensile strength = 700 MPa [48]), and G19-9NbSi (yield stress 460 MPa, ultimate tensile strength = 630 MPa [46]). The NiCr23Mo16 wire is used in highly aggressive environments. It is recommended for joining duplex and super duplex steels, stainless steels and nickel-based alloys [34]. The G19-9NbSi wire is successfully employed for welding unalloyed steels with the superalloy [20,30].

The preparation of the three-stitched joints, and the bevelling method of the steel sheet, are presented in Figure 3 and Figure 4.

The chamfering at the angle of 45° is intended to reduce the tendency of a joint to crack. In the welding process, an additional distance of 2 mm was assumed, Figure 3. This allowed for limiting the degree of mixing in the weld of base material chemical composition and welding wire during the welding process. The stitches laying order is presented in Figure 4.

In this case worth to notice various methods of bevelling were examined. Double bevel did not give incorrect results. The one-sided bevelling was used to equalise the conditions of heat distribution during welding.

The welding parameters were as follows: the electrode wire diameter was 1.2 mm, the arc voltage U = 21 V and the welding current was different in the root and the face layers, Figure 3. In the lower stitch arrangement, the I_3_ current ranged from 130 to 150 A, while the I_2_ current in the two upper layers ranged from 120 to 150 A. The welded sheets had dimensions of 800 mm × 200 mm × 8 mm, and the weld had a three-stitched character. In the MIG process, the following mixtures were used as shielding gases: 95% Ar-5% He and 90% Ar-10% He. The shielding gas flow rate was at the level of 15 L/min. The joint was made with variable tested speed V_3_: 200–230 mm/min (bottom layer) and V_1,2_: 220–270 mm/min (upper layer). MIG welding method (131) in the down position (PA) was selected according to the requirements of EN 15614-1 norm. Rotor joints were welded with direct current with a positive polarity on the electrode. Preheating was not applied.

### Tests Details

It was decided to verify the weldability of the joint made of Alloy 59 and S355J2W+N low-alloy steel. After producing welded joints using different parameters (two different electrode wires and two different shielding mixtures Ar-He), visual tests were carried out (PN-EN 970: 1999 standard [67]).

The tests aimed to assess the correctness of joints, identify incompatibilities in the form of cracks, and eliminate any incorrectly made connections. The analysis was expanded, including the results of non-destructive tests: penetration (PN-EN 571: 1999 standard [68]) and ultrasonic (PN-EN 1714: 2002 standard [69]). The test results were documented as macroscopic images (PN-EN 1321: 2000 standard [70]).

Connections that did not present macroscopic changes in the form of cold, hot and lamellar cracks were qualified for additional mechanical (bending, hardness, tensile tests) and microscopic observations. The joints presenting the best mechanical properties were subjected to microscopic inspection to examine the influence of welding parameters on the microstructure. The proposed evaluation results were the prerequisite for selecting parameters and processes required for manufacturing a mixed butt welded joint of a rotor structure.

The geometry of the hourglass specimen allows us to follow the requirements for fatigue tests, shown in American standards, i.e., ASTM-E468 [71] and ASTM-E466 [72]. This geometry is expressed by the shape of the measurement section and the value of the radius. These details were employed because the middle zone of the measurement section can be easily used for a weld, and this region can be easily subjected to loading without influencing the other specimen sections on results.

The specimens for the mechanical test were randomly selected from a pool of 20 specimens previously inspected for defects. In practical terms, these specimens were defect-free, i.e., identical in quality.

**Figure 5:**
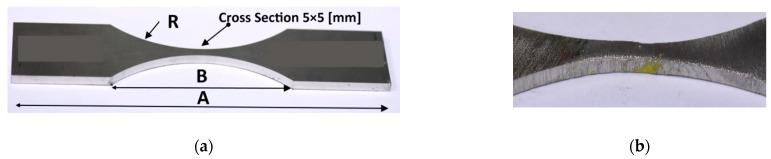

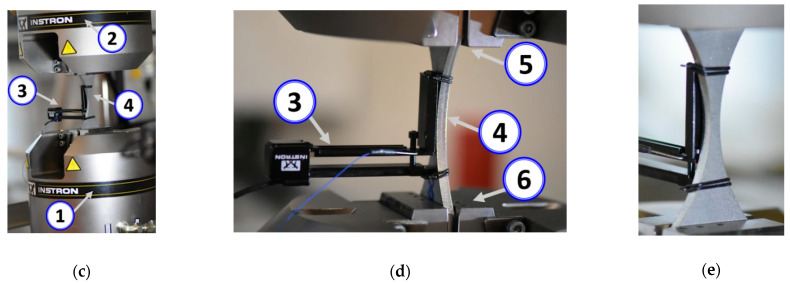
Details of the experiment: (**a**) Alloy 59 hourglass specimen as a base metal, (**b**) hourglass specimen with hybrid weld in the middle of the measurement section, (**c**) and the 2620-601 one-axial Instron extensometer in the gripping system of the 8802 Instron testing machine, 1, 2—hydraulic lower and upper grip (**c**–**e**) and, 3—the extensometer, 4—the specimen, 5, 6—flat jaw faces; A = 840 mm, B = 87 mm, R = 64 mm. The specimen is the author’s project proposed for weld examination under tensile force because this joint occurs in the middle measurement region (**b**).

Tensile tests were carried out considering the requirements of PN-EN ISO 6892-1: 2020 standard [73], at room temperature employing hourglass specimens and servo-hydraulic testing machines denoted by 8802 Instron, Figure 5. The testing machine was equipped with an alignment system to avoid the bending moment. Bluehill Instron software was used to elaborate stages of the tensile tests up to fracture. Specimens were directly mounted in hydraulic grips. Nominal dimensions of the specimens in the minimum cross-section were represented by the following values: 5 × 5 [mm], Figure 5a. A hybrid joint was located in the middle section of the measurement region and was directly subjected to loading. The testing machine was tuned at a close-loop feedback signal for the displacement velocity of 2 mm/min. Measurements of an axial strain were conducted using the extensometer technique using the 2620-601 Instron sensor, Figure 5b,c. This device has enabled capturing values of the axial strain at the gauge length equal to 50 mm. Concerning the range of the strain measurement, the extensometer has allowed collection results up to 10% strain, i.e., 5 mm. Therefore, the experimental programme has contained the stages for the extensometer removal at the value of elongation mentioned. The tested materials’ behaviour under a tensile force was not only recorded in the form of digital results but also it was expressed by photos from macro-photography techniques, collecting details of the measurement sections and fracture zones.

Once the joints were welded with the use of various parameters (two different electrode wires, three different shielding mixtures, different linear energy of the process), the visual (PN-EN 970: 1999 standard), penetrating (PN-EN 571: 1999 standard), macroscopic (PN-EN 1321: 2000 standard) and ultrasonic (PN-EN 1714: 2002 standard) tests were performed.

## 3. Results and Discussion

### 3.1. Weld Inspection, Hardness and Microstructure

Based on the inspection, it was found that:

- There is an occurrence of small cracks in joints made with G19-9NbSi austenitic wire using the following shielding gases: argon and Ar-10% He;
- No defects and inconsistencies for the B level (according to PN-EN ISO 5817: 2005 standard [74]) appeared in joints made with G19-9NbSi austenitic wire and the shielding compound of Ar-5% He;
- No defects or incompatibilities for the B level (according to PN-EN ISO 5817: 2005 standard) occurred after using NiCr23Mo16 electrode wire and a tested shielding gas mixture (Ar-5% He, Ar-10% He).

For the further approaches (bending test), only joints without defects and incompatibilities were selected. The joints manufactured at G19-9NbSi austenitic electrode wire and the Ar-10% He shielding mixture were not satisfactory for the experimental procedure. The use of austenitic wire during the MIG welding method (131) did not produce connections characterised by the desired level of quality B (according to PN-EN ISO 5817: 2005). Due to unsatisfactory results of the external examination of joints and cracks, connections made with austenitic wire were rejected. For the other joints examined, a bending test was carried out by the PN-EN ISO 5173: 2010 standard [75]. For the tests, a specimen with thickness a = 8 mm, width b = 10 mm, mandrel d = 32 mm, roller distance 54 mm and bending angle of 180° was used. Five measurements of the bending test were made from the face and the root side of the weld. Only when the G19 -9NbSi austenitic electrode wire and the Ar-5% He mixture were used cracks in the weld were observed at a bending angle above 130°. As a result, it can be concluded that the adopted welding parameters made with austenitic wire will have lower performance properties than welds made with the same welding current–voltage parameters and a different welding wire.

When a nickel-based electrode wire NiCr23Mo16 was used (together with the two tested shielding gases), no cracks or other incompatibilities were found in the tested specimens. Both the macroscopic observations and the bending test results showed that the welded joints were made accurately, the selected welding parameters were correct, and the adopted bevelling angle and NiCr23Mo16 wire allowed us to obtain joints with the required quality level.

Due to the occurring defects in the joint made with the use of austenitic electrode wire G19-9NbSi, in the further part of the study, it was decided to analyse only those joints made with the nickel-based wire NiCr23Mo16. Tests of immediate tensile strength were carried out. They were performed on a ZWICK 100N5A strength-testing machine.

Data analysis (Table 2) shows that the welds are made correctly. All joints have comparable mechanical properties (YS above 320 MPa; UTS above 520 MPa). The table data also expresses that welds made with lesser linear energy have higher YS values (lower current, higher speed). Joint yield stress should be above 355 MPa (in accordance with the symbol and requirements of S355JR+N steel). Such a high value of yield stress can be obtained when welding with lower linear energy and the use of a shielding compound Ar-5% He. It can also be observed that the shielding mixture of 95% Ar-5% He is the most advantageous due to the highest value of ultimate tensile strength (553 MPa).

The introduction of helium into the mixture in a small amount affects the shape of the weld, increasing its concavity, which according to the literature data [22], is very beneficial. Helium has a higher heat transfer coefficient than argon. As a result, adding more helium to an argon mix may affect the grinding of grain in the weld. The helium content in the argon mixes up to 10% might be considered ineffective, as it does not guarantee yield stress of 355 MPa and, at the same time, does provide a further increase in joint strength. It was observed that in all the studied cases, welding with lower linear energy is more advantageous. The use of NiCr23Mo16 electrode wire together with the Ar-5% He gas mixture is the most appropriate, as it allows us to obtain the highest values of yield stress (365 MPa) and ultimate tensile strength (550 MPa) of the joint.

**Figure 6:**
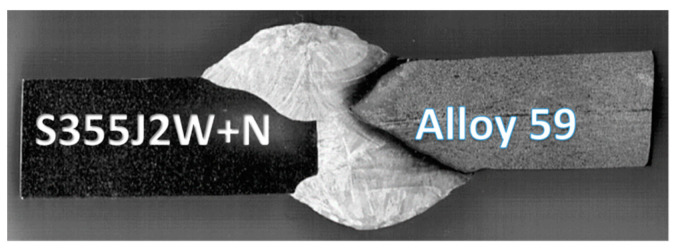
Macroscopic photos of the 8 mm thick joint made of rust-resistant S355J2W+N steel (left side of the weld) and Alloy 59 (right side of the weld).

It is worth noticing that, with respect to the paper’s aim, the SEM method was not used, but the macro-photography technique (Figure 6) was applied to collect details of the joint manufactured. This has enabled us to follow the weld quality and qualified it for the mechanical tests and microstructural observations.

Vickers steel hardness amounted to 185 MPa, whereas Alloy 59 hardness was equal to 375 MPa. Table 3 presents the results of hardness tests in the heat-affected zone from both welded sides and the weld hardness in the six tested joints (electrode wire based on NiCr23Mo16 nickel alloy, two gas shielding mixtures and two different welding linear energies). The table data shows that an increase in the helium content in the Ar-He mix has a direct influence on the increase in hardness value. The most favourable results were obtained for joints made using an Ar-5% He mixer. An increase in the welding linear energy does not cause any noticeable changes in the hardness of the weld, Table 3.

The analysis of Table 3 shows that the joint is made correctly. Hardness test results on the Vickers method show that obtained joints using gas mixture Ar-5% He is more beneficial (because the hardness difference in tested join zones is the lowest), Figure 3 and Figure 6.

Based on the analysis of table data, the following welding parameters were finally selected for making joints for all further tests:

- Electrode wire: NiCr23Mo16 wire;
- Gas mixture: Ar-5% He, current and welding speed: I_3_ = 110, V_3_ = 230 mm/min, I_2_ = 120 A, I_1_ = 130 A, V_1,2_ = 270 mm/min.

**Figure 7:**
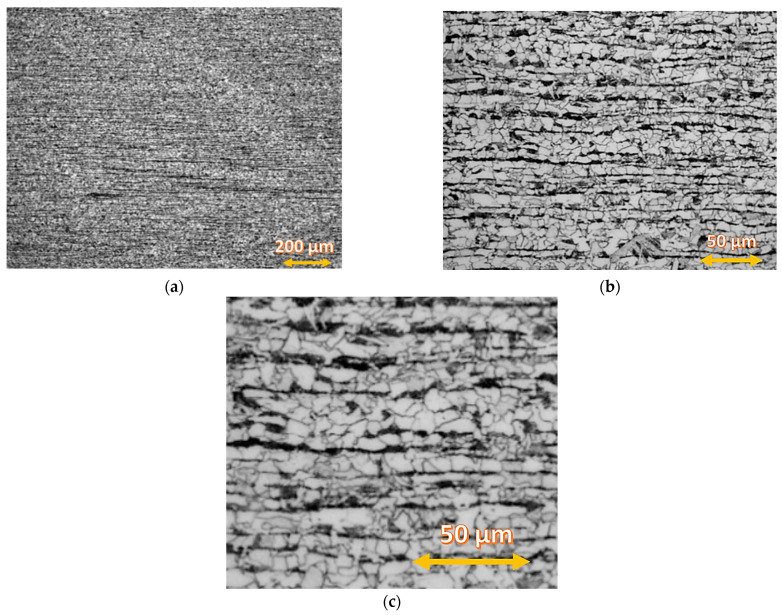
(**a**–**c**) S355J2W+N steel, ferritic–pearlitic microstructure under different magnification.

**Figure 8:**
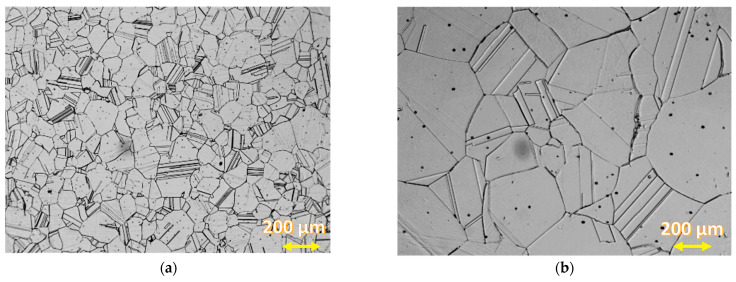
Alloy 59—(**a**,**b**) austenitic microstructure under different magnification.

The fuse was complete with a clear fusion line. This indicated that both the bevelling method and the welding parameters were selected properly. Production of a welded joint is not easy because of the different microstructures of both materials and other heat transfer coefficients. The thermal conductivity of steel amounts to 60 W/(m·K), whereas the thermal conductivity of the alloy is at the level of 90 W/(m·K). Steel S355J2W+N has a ferritic–pearlitic microstructure (Figure 7), while Alloy 59 has a single-phase austenitic microstructure with good solubility of alloying elements in the FCC nickel network, Figure 8.

The microstructure observations were performed on the LM (light microscopy) observation under various magnifications. The specimen was digested in Adler’s A11 reagent. Figure 8 shows the ferritic–pearlitic microstructure of the base material.

**Figure 9:**
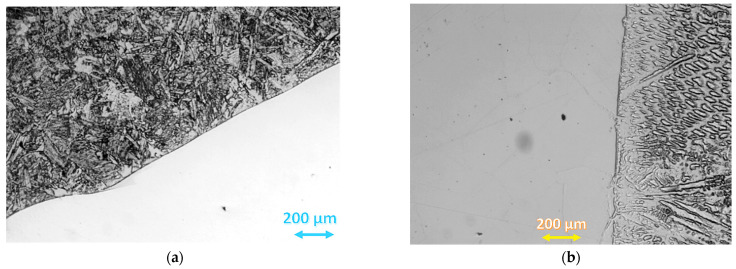
Mixed joint fusion line: (**a**) fusion line from the S355J2W+N steel side, (**b**): fusion line from the Alloy 59 side.

Figure 9a shows the microstructure of the joint from the S355J2W+N steel side, whereas Figure 9b shows the section of the fusion line from the Alloy 59 side.

The analysis shows the base material adjacent to the fusion line has a high content of coarse ferrite and MAC phases (martensite, residual austenite, carbides), whereas in the superalloy base material, a two-phase austenitic–ferritic microstructure appeared. The observation of the weld on a micro-scale confirms that the joint was manufactured correctly. In addition to the comment on the fusion line, it was decided to verify the joint microstructure (under various magnifications) in the central part of the weld, Figure 9.

Figure 8 shows a single-phase, semi-similar austenitic microstructure of Alloy 59. The microstructure of both base materials changes when approaching the weld.

**Figure 10:**
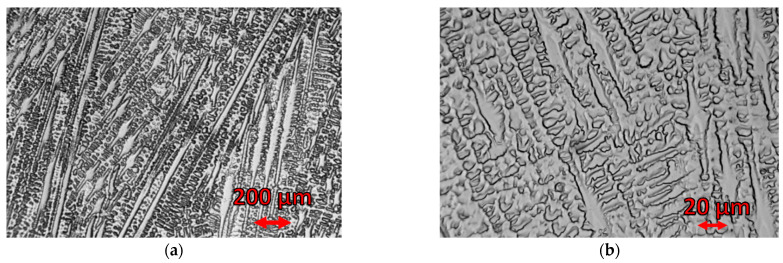
The microstructure of the mixed joint (**a**) central part made of S355J2W+N steel and Alloy 59. (**b**) enlargement of the selected area shown in (**a**).

Figure 10 shows an austenitic–ferritic microstructure with a favourable dendritic cell formation. Based on the calculations of iron (about 30%), nickel (40%) and chromium equivalents, as well as the equilibrium graph (Figure 1), it can be concluded that the weld microstructure should contain about 95% of austenite and about 5% of delta ferrite. The dendritic and cellular natures of austenite translate into the excellent plastic properties of a joint.

The obtained results have allowed us to select the process parameters with respect to the high quality of the joint. It was decided to perform the mechanical resistance of the hybrid joint in detailed tests.

Joints should be made of low-alloy steel with low-alloy steel and Alloy 59 with Alloy 59 to determine whether the hybrid joints will have mechanical properties (as a result or similar) to one of the joints. The same parameters of the welding process were used to make all the joints in that part of the investigation:

- Electrode wire: NiCr23Mo16 wire,
- Gas mixture: Ar-5% He,
- Current and welding speed: I_3_ = 110, V_3_ = 230 mm/min, I_2_ = 120 A, I_1_ = 130 A, V_1,2_ = 270 mm/min.

### 3.2. The Base Metal and MIG Hybrid Weld under Tensile Force

**Figure 11:**
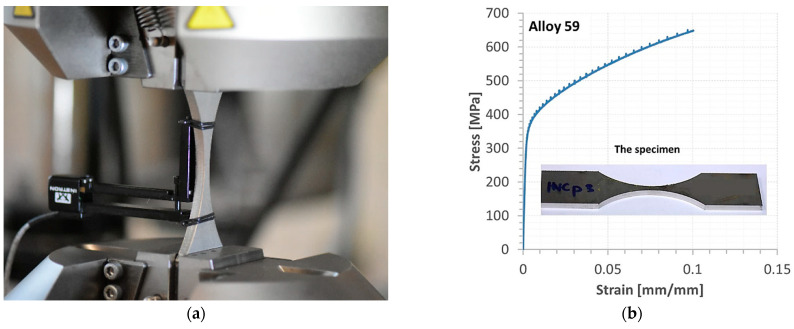
Alloy 59 specimen before the tensile test (**a**); a part of the stress–strain characteristic of the alloy joint up to the extensometer removing (**b**).

**Figure 12:**
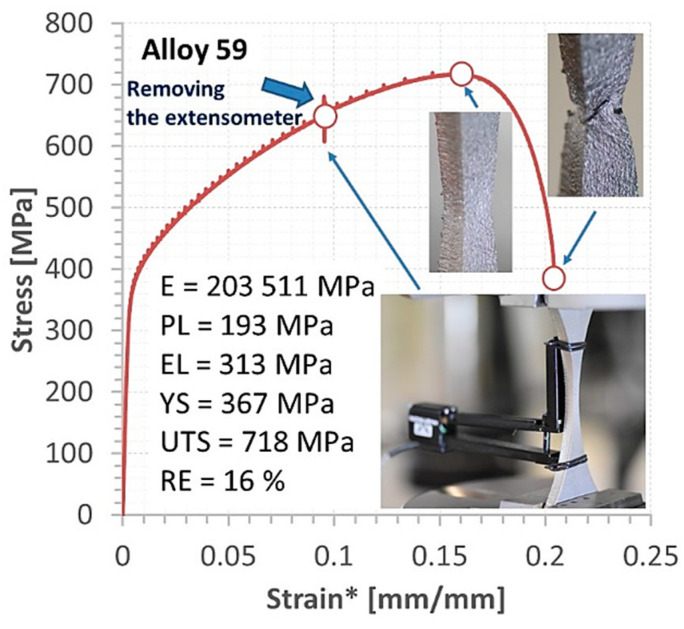
Stress–strain curve of the Alloy 59, *—strain determined based on data from Figure 11 and values of displacement.

**Figure 13:**
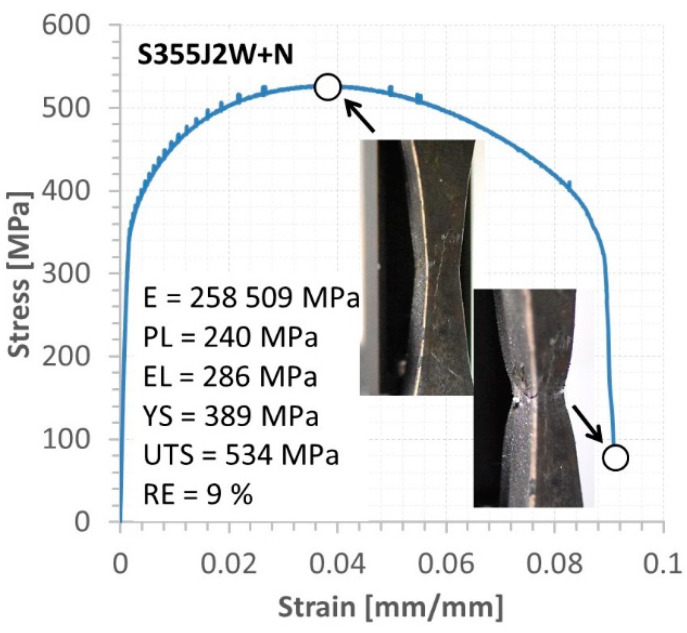
Tensile characteristics and mechanical parameters of S355J2W+N steel.

**Figure 14:**
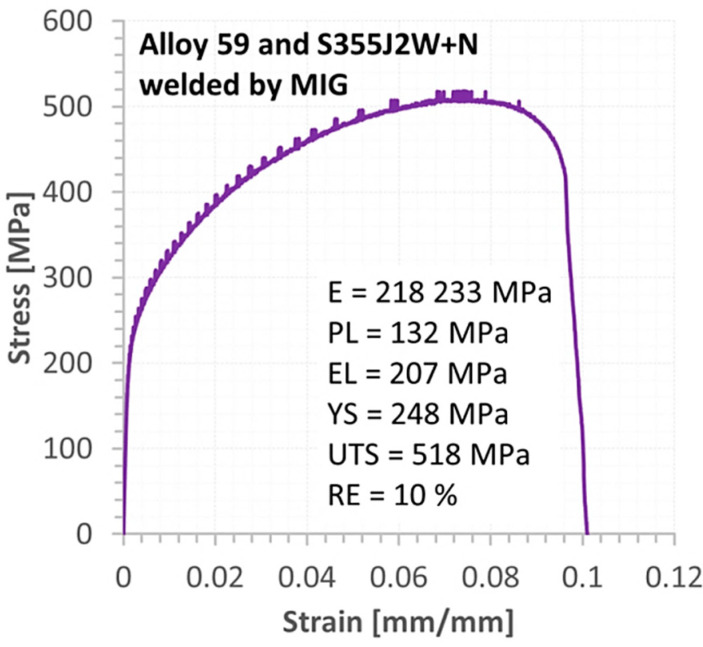
Tensile curve of the MIG weld for Alloy 59 and S355J2W+N steel.

**Figure 15:**
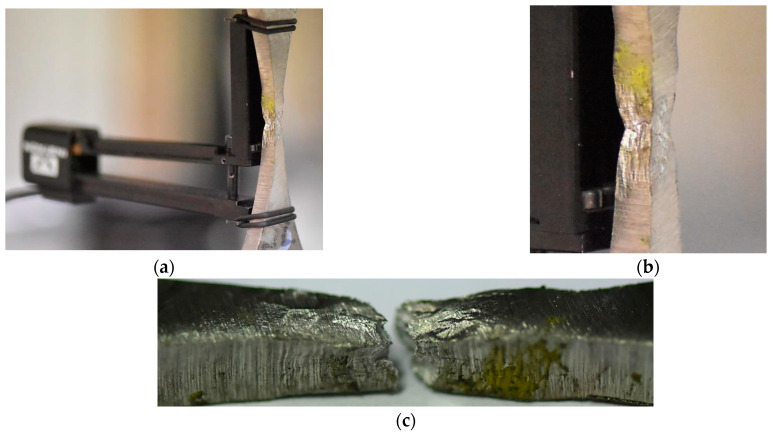
The MIG weld of Alloy 59 and S355J2W+N steel: (**a**,**b**) at the fracture, (**c**) after the fracture.

The behaviour of the base metals and weld was expressed by the stress–strain relationship up to fracture and mechanical parameters from an elastic and elastic–plastic state, Figure 11, Figure 12, Figure 13, Figure 14 and Figure 15. In the case of Alloy 59 (as the base metal), the value of axial strain was the biggest one, and it exceeded its limited value related to the extensometer used, Figure 11b. Therefore, for the experimental way, values of strain were calculated based on the values of strain from the extensometer and displacement from a linear sensor of the testing machine, comparing the stress–strain relationship collected at both measurement sensor activities.

The final results of this approach are presented in the form of the tensile curve and mechanical parameters of Alloy 59, Figure 12. It can be noticed that the superalloy has obtained a wide range of elastic regions between values of proportional limit and elastic limit, i.e., represented by almost 100 MPa. This value follows the 50% value of the proportional limit and takes a significant meaning in the elastic behaviour of the joint tested. The other section of the tensile curve is dominant, expressing a wide range of hardening represented by 405 MPa. This hardening can be directly taken to the engineering approach concerning construction safety because of the operation state with plastic deformation, the final fracture can be noticed by many measurement techniques, enabling to avoid unexpected failure. Analysing the fracture zone allows for distinguishing the stress component directly connected with the weld degradation. In this case, the shear stress created the final cracking, Figure 12.

In the case of the S355J2W+N steel, the elastic region was limited by a value close to 290 MPa, while the elastic–plastic with hardening is differenced by 250 MPa, Figure 13. These values have enabled us to write as follows: the elastic and elastic–plastic sections of the tensile curve are significant in the weld behaviour. The last region of the characteristic directly expressed the unstable behaviour of the joint, reflecting the neck effect at an extensive range of strain and stress, i.e., 6% (66% of the final plastic deformation) and 454 MPa, respectively. It means the inspection of a component having this type of weld should be carried out carefully and more often than in the case of a typical one because avoiding the stage related to the necking.

The behaviour of the mixed Alloy 59 and S355J2W+N steel weld (Figure 14) reflected that in the comparison to the proportional section, the elastic region was represented by smaller values compared to data of the welded base metals: Figure 12 and Figure 13.

The elastic–plastic part of the curve up to the ultimate tensile curve was the critical section of the characteristic considered. This was denoted by the value of a stress range equal to 311 MPa and 80% of the plastic deformation. It can be concluded the tested weld response with respect to plastic features is very similar to the MIG joint made of steel. The fracturing of the hybrid weld was mixed, containing brittle–ductile features created by both stress components, i.e., axial and shear, Figure 15.

**Figure 16:**
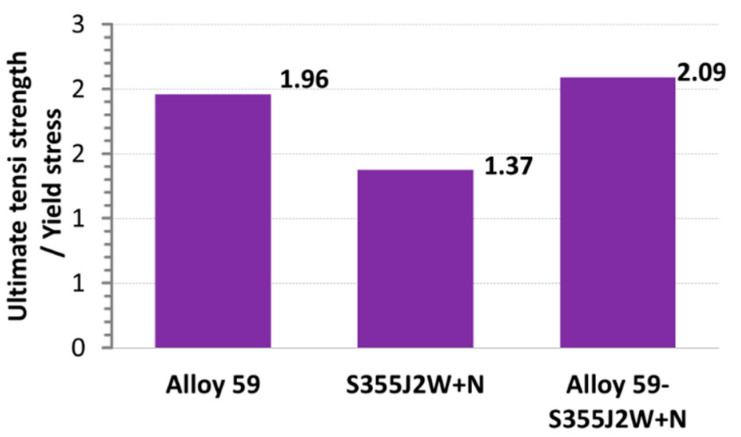
Proportion between ultimate tensile strength and yield stress of the base metals (Alloy 59, S355J2W+N) and the mixed joined. Values of the mechanical parameters are directly taken from Figure 13, Figure 14, Figure 15 and Figure 16.

**Figure 17:**
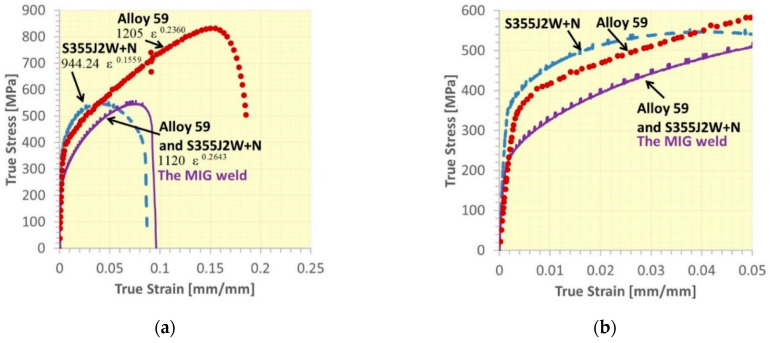
True stress–true strain curves of the Alloy 59, S355J2W+N steel and their MIG joint: (**a**) full characteristics, (**b**) the initial sections of the tensile curves from (**a**).

Comparing data for the weld types, represented by ultimate tensile strength and yield stress, it can be observed the proportion of the mechanical parameters considered for the alloy and hybrid welds is very similar, while in the case of the steel, it is 30% lower, Figure 16. This indicates the hardening curves can have very similar features, besides the weld types being enormously different. It was checked and confirmed using a power law (*σ = Kε^n^*, *K*–strength coefficient*, n*—strain hardening exponent, [41,42], calculating all coefficients of the equation at the true stress–true strain relationship ranged by yield stress (YS) and ultimate tensile strength (UTS), Table 4, Figure 17. As can be noticed, in the case of the alloy and the welds for alloy steel the values of the coefficients are very similar. It means the stress–strain relationship for the Alloy 59 and the weld being its combination with the S355J2W+N steel has a similar path for its shape, indicating the examined regions express almost the same response on the tensile and by this, they can be analysed using constitutive equations having very similar values of coefficients.

**Figure 18:**
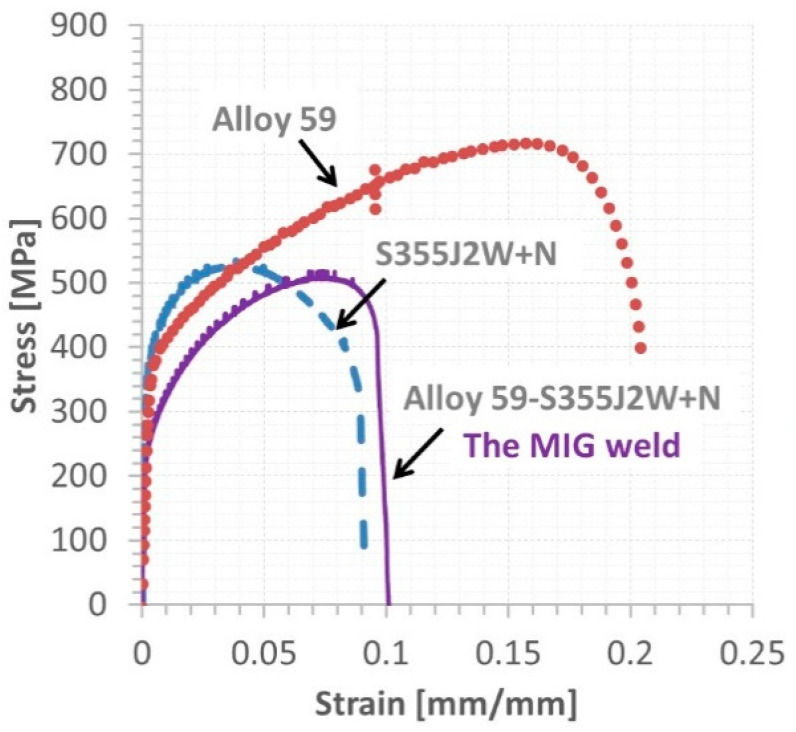
Engineering tensile characteristics of the Alloy 59, S355J2W+N steel and their MIG joint from Figure 13, Figure 14 and Figure 15.

**Figure 19:**
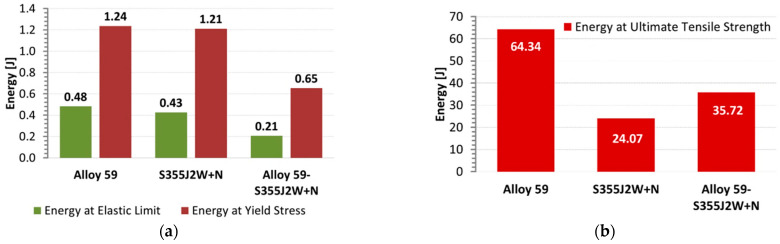
Energy at mechanical parameters for Alloy 59, S355J2W+N steel and the mixed weld calculated based on the tensile curves from Figure 13, Figure 14 and Figure 15. (**a**) for the elastic limit and yield stress (**b**) for the ultimate tensile strength.

Some conclusions on the behaviour of the weld tested can also be captured based on the engineering tensile curves as data for the typical approaches for analysis of mechanical resistance of joints and welded components under various types of loading using theoretical and numerical stages, Figure 18. They are expressed by values of a relative strain as well as values of ultimate tensile strength. As it can be noticed, in the case of the steel and its connection with alloy, a reduction of elongation was expressed by 50% compared to data for the alloy, while the ultimate tensile strength was only lowered by 30%. Moreover, the hybrid joint can be called the weakest weld because this zone has reached the smallest values of proportional limit, elastic limit, yield stress and ultimate tensile strength. This result is confirmed in values of energy related to elastic limit and yield stress, Figure 19a, while the energy values at the ultimate tensile strength of the hybrid joint were not the smallest ones, Figure 19b. Nevertheless, in engineering practice, a material behaviour at an elastic state plays an essential role in modelling, designing and operating, therefore at the smallest values of data from the state considered and the 33% difference in energy value for the ultimate tensile strength of the hybrid joint.

**Figure 20:**
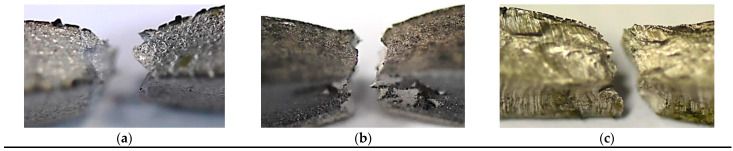
Geometrical differences in fracture zones at elastic–plastic deformation, with different degradation types: (**a**) multi-plane—Alloy 59, (**b**) double-plane—S355J2W+N steel and their MIG connection—one-plane (**c**).

Other details on the behaviour of the tested regions can be captured in the analysis of fracture regions, Figure 20. They are connected with the geometrical features of the zones considered because changes in fracturing sections reflect variations in stress state components and enable formulating the conclusion on the role of stress type in the zones’ degradation. Looking at the fracture region of Alloy 59 (Figure 20a), the multi-planar degradation can be indicated as the main feature due to the loading type used. In contrast, in the case of S355J2W+N steel (Figure 20b) and the steel alloy (Figure 20c) joint, the region is represented by a one-fracture plane. Therefore, the following sentences can be formulated:

(a) Reorientation of axial and shear stress components follow the degradation of the Alloy 59 as well as differences in their values as stress state components;
(b) In the case of the weld manufactured by means of Alloy 59 and S355J2W+N steel, the proportion between axial and shear stress can be indicated as a constant because the fracturing is represented by one fundamental region.

For further experiments, it is worth focusing on SEM approaches to the fracture region because more details on the weld degradation can be collected. These details on the weld can be directly used for damage mechanics for the weld behaviour description concerning damages due to monotonic tensile. Moreover, the degradation mechanism can be more clearly presented, and a scheme for the damage-type features is possibly easily presented.

Another important stage for the further examination of the proposed weld technology and the structural materials can be connected with quantitative determination because it enables us to follow an extended measurement uncertainty employing a calliper and testing machine accuracy as well as an error related to a tested object mounting. Taking those details, the uncertainty of the individual components of the experiment can be covered. Next, expanded and complex uncertainties can be resolved. This approach will avoid significant mistakes, and following the quality of the welds manufactured using different material types can be discussed very precisely.

## 4. Novelty and Application

The novelty and application of the welding technology and the testing method can be presented as follows:

Novelty

- The MIG process at the determined parameters can be directly used for mixed joint manufacturing;
- The welding process does not require additional devices or systems, i.e., cooling or heating;
- The hourglass specimen with a weld in its middle region of a measurement section is very useful for determining the joint quality;
- For mixed joint quality, the fundamental features of the joint such as stress–strain characteristics, mechanical parameters and hardening curves for analytical and FEA approaches are determined.

Application

- Improvement of welding technology for other mixed metal joints;
- Power plant industry for operational conditions at elevated temperatures and inspections for replacing selected components due to failure;
- Analytical and numerical approaches for superalloy and steel welding using the collected results;
- Forecasting service life using the determined mechanical parameters of the joint.

## 5. Summary

The analysis of the obtained test results enabled us to formulate as below:

- It is possible to make a correct mixed joint made of S355J2W + N steel and Alloy 59 using the MIG process without welding defects and incompatibilities.
- The MIG connection technology with one-side bevelling and using NiCr23Mo16 nickel-based electrode wire and Ar-5% He shielding gas is the correct choice.
- The parameters of the welding technology for joining superalloy (Alloy 59) and S355 steel:
  o Electrode wire: NiCr23Mo16 wire,
  o Gas mixture: Ar-5% He, current and welding speed: I_3_ = 110, V_3_ = 230 mm/min, I_2_ = 120 A, I_1_ = 130 A, V_1,2_ = 270 mm/min.
- The mixed weld had excellent mechanical properties: yield stress (248 MPa) and ultimate tensile strength (518 MPa) values, which means the joint can be applied to rotor structural elements.
- In the case of the superalloy and mixed joint, the hardening sections of the tensile curves were very similar in shape, and digital results represented almost the same values of power law coefficients.
- The fracturing of the steel and mixed weld was expressed by the one fundamental decohesion region, which has reflected the constant proportion of values of axial and shear stress components up to the separation.

## Figures and Tables

**Table 2:** Mechanical properties of the tested joints manufactured using the NiCr23Mo16 wire, YS- yield stress, UTS—ultimate tensile strength.

Parameters of the Lower Stitch Pattern	Parameters of the Upper Stitch Pattern	Shielding Gases	YS, [MPa]	UTS, [MPa]	UTS/YS
U = 21 V I_3_ = 110 V_3_ = 230 mm/min	U = 21 V I_2_ = 120 A I_1_ = 130 A V_1,2_ = 240 mm/min	Ar-5% He	365	553	1.52
		Ar-10% He	346	547	1.58
U = 21 V I_3_ = 120 V_3_ = 210 mm/min	U = 21 V I_2_ = 140 A I_1_ = 150 A V_1,2_ = 220 mm/min	Ar-5% He	341	537	1.57
		Ar-10% He	333	534	1.60

**Table 3:** Hardness test results on Vickers method, HAZ—heat-affected zone.

Parameters of:			Hardness in Point: 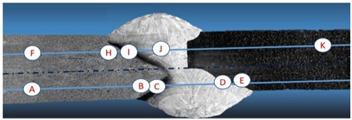									
The Lower Stitch Pattern	The Upper Stitch Pattern	Shielding Gases										
			A	B	C	D	E	F	H	I	J	K
U = 21 V I_3_ = 110 V_3_ = 230 mm/min	U = 21 V I_2_ = 120 A I_1_ = 130 A V_1,2_ = 270 mm/min	Ar-5% He	371	345	333	250	224	376	347	330	286	186
		Ar-10% He	378	352	346	248	231	378	354	345	298	184
U = 21 V I_3_ = 120 V_3_ = 200 mm/min	U = 21 V I_2_ = 140 A I_1_ = 150 A V_1,2_ = 220 mm/min	Ar-5% He	372	346	329	246	218	371	353	330	291	188
		Ar-10% He	380	355	341	251	227	375	358	339	296	185

**Table 4:** Power law equations for the Alloy 59, S355J2W+N steel and their MIG joining for the true stress–true strain curves shown in Figure 18.

**Structural Materials**		
Alloy 59 (base metal)	S355J2W+N (base metal)	Alloy 59—S355J2W+N (MIG weld)
**Power law**		
1205 ε ^0.2360^	944.24 ε ^0.1559^	1120 ε ^0.2643^

## Data Availability

Not applicable.

## References

[1] Azadian S. (2004). Aspects of Precipitation in the Alloy Inconel 718. Ph.D. Thesis.

[2] Scarlin R.B. (2010). Bauteil für eine Hochtemperaturdampfturbine Sowie Hochtemperaturdampfturbine. Germany Patent.

[3] James A.W., Rajagopalan S., Shirzadi A., Jackson S. (2014). 1—Gas Turbines: Operating Conditions, Components and Material Requirements. Structural Alloys for Power Plants.

[4] Nissley N.E., Collins M.G., Guaytima G., Lippold J.C. (2003). Development of the Strain-to-Fracture Test for Evaluating Ductility-Dip Cracking in Austenitic Stainless Steels and Ni-Base Alloys. Weld. Res..

[5] Prakash S., Shirzadi A., Jackson S. (2014). Development of Advanced Alloys with Improved Resistance to Corrosion and Stress Corrosion Cracking (SCC) in Power Plants. Structural Alloys for Power Plants.

[6] Scarlin R.B. (2005). Thermally Loaded Component, and Process for Producing the. Component. Patent.

[7] Barnes M., Jones R.L., Abson D.J., Gooch Twi T.G., Strang A. (2000). Welding and Fabrication of High Temperature Components for Advanced Power Plant. Materials for High Temperature Power Generation and Process Plant Applications.

[8] Starr F., Shirzadi A., Jackson S. (2014). High Temperature Materials Issues in the Design and Operation of Coal-Fired Steam Turbines and Plant. Structural Alloys for Power Plants.

[9] Ohji M.H. (2017). Steam turbine cycles and cycle design optimization. Adv. Steam Turbines Mod. Power Plants.

[10] Keller S., Scarlin B., Staubli M., Vanstone R. (2009). Method of Welding Together Two Thermally Differently Loaded Parts e.g., for Turbo-Machine, Requires Initially Positioning Inter-Layer on Connection Surface of Second Part. JP Patent.

[11] Keller S. (2002). Method for Welding Together Two Parts Which Are Exposed to Different Temperatures, and Turbomachine Produced using a Method of This Type. U.S. Patent.

[12] Scarlin R.B. (2005). Welded Rotor used for Thermal Machine and Method of Manufacturing the Rotor. JP Patent.

[13] Lippod J.C., Kiser S.D., Dupont J.N. (2009). Welding Metallurgy and Weldability of Nickel-Base 19 Alloys.

[14] Herderick E. (2011). Additive manufacturing of metals: A review. Mater. Sci. Technol. Conf. Exhib..

[15] Frazier W.E. (2014). Metal additive manufacturing: A review. J. Mater. Eng. Perform..

[16] Benson T.H., Shoeppner G.A. (2002). Accelerating materials insertion by evolving materials qualification-transition paradigm. AMMITAC Q..

[17] Bai Z.F., Li G.Z., Wang C., Wang L., Ahang Z. (2011). Microstructure and Mechanical Property of the Welded Joints of S355J2W+N Steel. J. Jilin Univ. (Eng. Technol. Ed.).

[18] Pałubicki S., Karpiński S. (2015). Linear energy impact on formation of hot cracks in the welding process of S355J2WP by 135 method. Weld. Technol. Rev..

[19] Chu T., Xu H., Li Z., Lu F. (2019). Investigation of intrinsic correlation between microstructure evolution and mechanical properties for nickel-based weld metal. Mater. Des..

[20] Baufeld B. (2012). Mechanical properties of Inconel 718 parts manufactured by shaped metal deposition (SMD). JMEP.

[21] Suppliers of Speciality Metal Alloys and Nickel Alloys, Alloy C276. www.neonickel.com/pl/alloys/stopy-niklu/alloy-c276/.

[22] Schaeffler A.L. (1949). Constitution diagram for stainless steel weld metal. Met. Prog..

[23] Ni-Cr-Fe Phase Diagram. https://blog.utp.edu.co/metalografia/7-aceros-inoxidables/.

[24] Mageshkumar K., Arivazhagan C., Kuppan P. (2019). Studies on the effect of filler wires on micro level segregation of alloying elements in the alloy 617 weld fusion zone. Mater. Res. Express.

[25] Fujita M., Nishitani S., Nakatake Y., Doi M., Kudou S., Shono K., Asao Y., Kuribayashi M. (2007). Welding set of metal member including permanent magnet and welding method thereof, as well as electric rotating machine. JP Patent.

[26] Nazmy M., Gerdes P.C., Kuenzler A. (2010). Welding Additive Material. US Patent.

[27] Balbach W.M., Keller S., Redecker R. (2001). Process for Welding High Alloyed Heat-Resistant Martensitic/Ferritic Steels or Super Alloys Comprises Plating a First Component and Selectively also a Second Component, Optionally Heat Treating, Joint Welding and Annealing. Germany Patent.

[28] Nickel-Chromium (Ni-Cr) Phase Diagram. https://www.calphad.com/iron-nickel.html.

[29] Makowska K., Brodecki A., Mackiewicz S., Kowalewski Z.L. (2018). Damage development of Inconel 718 due to laboratory simulated creep. J. Theor. Appl. Mech..

[30] Xiao L., Chen D.L., Chaturvedi M.C. (2004). Shearing of γ′′ precipitates and formation of planar slip bands in Inconel 718 during cyclic deformation. Scr. Mater..

[31] Jeffrey W., Sowards J.L.C., Hashmi S. (2014). Weldability of nickel-base alloys In Comprehensive Materials Processing.

[32] Zhao X., Chen J., Lin X., Huang W. (2008). Study on microstructure and mechanical properties of laser rapid forming Inconel 718. Mater. Sci. Eng. A..

[33] Walsh S.M., Smith J.P., Browne E.A., Hennighausen T.W., Malouin B.A. Practical concerns for adoption of microjet cooling. Proceedings of the ASME Proceedings 2018 Power Electronics, Energy Conversion, and Storage.

[34] Dinda G.P., Dasgupta A.K., Mazumder J. (2009). Laser aided direct metal deposition of Inconel 625 superalloy: Microstructural evolution and thermal stability. Mater. Sci. Eng. A.

[35] Murr L.E., Martinez E., Gaytan S.M., Ramirez D.A., Machado B.I., Shindo P.W., Martinez J.L., Medina F., Wooten J., Ciscel D. (2011). Microstructural architecture, microstructures, and mechanical properties of a nickel-base superalloy fabricated by electron beam melting. Metall. Trans. A.

[36] Han-Jin J.K., Minjung K., Phaniraj P., Jin-Yoo S., Park E.S., Kim D.-I., Hong S.T., Han H.N. (2018). Microstructure and mechanical properties of friction stir welded and laser welded high entropy alloy CrMnFeCoNi. Met. Mater. Int..

[37] Shuangming Y.L., Yang L.L., Zhong H. (2018). Microstructure and properties of twinned dendrites in directionally solidified A356 alloy. Mater. Sci. Eng. A.

[38] Ajasekhar K., Harendranath C.S., Raman R., Kulkarni S.D. (1997). Microstructural evolution during solidification of austenitic stainless steel weld metals: A color metallographic and electron microprobe analysis study. Mater. Charact..

[39] Ferreira F.A., Paradela K.G., Junior P.F., Júniora Z.A., Garci A. (2017). Phase-field simulation of microsegregation and dendritic growth during solidification of hypoeutectic Al-Cu alloys. Mater. Res..

[40] Tu S., Ren X., He J., Zhang Z. (2020). Stress–strain curves of metallic materials and post-necking strain hardening characterization: A review. Fatigue Fract. Eng. Mater. Struct..

[41] Kucharski S., Mróz Z. (2007). Identification of yield stress and plastic hardening parameters from a spherical indentation test. Int. J. Mech. Sci..

[42] Cooper D. (2015). Sheet Metal Forming 2.810.

[43] Hiremath P.S., Sadashivappa A., Pattan P. (2015). Analysis and characterization of dendrite structures from microstructure images of material. IJRET Int. J. Res. Eng. Technol..

[44] Roylance D. (2001). Stress-Strain Curves.

[45] Product Catalogue, Lasting Connections, Voestalpine Böhler Welding. Voestalpine Böhler Welding GmbH manual, January 2019, 671p. www.voestalpine.com/welding.

[46] BÖHLER SAS 2-IG (Si), Solid Wire High-Alloyed, Stainless, 03/2014; 1p. www.voestalpine.com/welding.

[47] Wen Y., Gao J., Narayan R.L., Wang P., Zhang L., Zhang B., Ramamurty U., Qu X. (2023). Microstructure-property correlations in as-built and heat-treated compositionally graded stainless steel 316L-Inconel 718 alloy fabricated by laser powder bed fusion. Mater. Sci. Eng. A.

[48] Raj S., Biswas P. (2022). Mechanical and microstructural characterizations of friction stir welded dissimilar butt joints of Inconel 718 and AISI 204Cu austenitic stainless steel. Mater. Charact..

[49] Anuradha M., Das Vemulapalli D., Cheepu M. (2022). Effect of filler materials on dissimilar TIG welding of Inconel 718 to high strength steel. Mater. Today Proc..

[50] Mortezaie A., Shamanian M. (2014). An assessment of microstructure, mechanical properties and corrosion resistance of dissimilar welds between Inconel 718 and 310S austenitic stainless steel. Int. J. Press. Vessel. Pip..

[51] Ravikiran K., Das G., Kumar S., Singh P.K., Sivaprasad K., Ghosh M. (2019). Narrow gap welding of low alloy and austenitic stainless steels using different Inconel alloys: Comparison of microstructure and properties. Mater. Res. Express.

[52] Jang C., Lee J., Kim J.S., Jin T.E. (2008). Mechanical property variation within Inconel 82/182 dissimilar metal weld between low alloy steel and 316 stainless steel. Int. J. Press. Vessel. Pip..

[53] Handa V., Goyal P., Sehgal S. (2020). Review of joining inconel alloys through microwave hybrid heating and other techniques. Mater. Today Proc..

[54] Li Y.-F., Hong S.-T., Choi H., Han H.N. (2019). Solid-state dissimilar joining of stainless steel 316L and Inconel 718 alloys by electrically assisted pressure joining. Mater. Charact..

[55] Müller R., Hengst P., Biermann H., Buchwalder A. (2022). Development of a basic technology for multilayer electron beam cladding of Inconel 718 nickel-based alloy onto an austenitic stainless steel. CIRP J. Manuf. Sci. Technol..

[56] Mishra N.K., Shrivastava A. (2023). Improvement in strength and ductility of rotary friction welded Inconel 600 and stainless steel 316L with Cu interlayer. CIRP J. Manuf. Sci. Technol..

[57] Navarro M., Matar A., Diltemiz S.F., Eshraghi M. (2022). Development of a low-cost wire arc additive manufacturing system. J. Manuf. Mater. Process..

[58] Tomota Y., Daikuhara S., Nagayama S., Sugawara M., Ozawa N., Adachi Y., Harjo S., Hattori S. (2014). Stress Corrosion Cracking Behavior at Inconel and Low Alloy Steel Weld Interfaces. Metall. Mater. Trans. A Phys. Metall. Mater. Sci..

[59] Borgmann C., Dumstorff P., Kern T.U., Almstedt H., Niepold K. Integrated weld quality concept: A holistic design approach for steam turbine rotor weld joints. Proceedings of the ASME Turbo Expo 2015: Turbine Technical Conference and Exposition.

[60] Zhu M.L., Xuan F.Z. (2010). Correlation between microstructure, hardness and strength in HAZ of dissimilar welds of rotor steels. Mater. Sci. Eng. A.

[61] Nivas R., Das G., Das S.K., Mahato B., Kumar S., Sivaprasad K., Singh P.K., Ghosh M. (2017). Effect of Stress Relief Annealing on Microstructure & Mechanical Properties of Welded Joints Between Low Alloy Carbon Steel and Stainless Steel. Metall. Mater. Trans. A Phys. Metall. Mater. Sci..

[62] Zhu Z.Y., Liu Y.L., Gou G.Q., Gao W., Chen J. (2021). Effect of heat input on interfacial characterization of the butter joint of hot-rolling CP-Ti/Q235 bimetallic sheets by Laser + CMT. Sci. Rep..

[63] Liang L., Xu M., Chen Y., Zhang T., Tong W., Liu H., Wang H., Li H. (2021). Effect of welding thermal treatment on the microstructure and mechanical properties of nickel-based superalloy fabricated by selective laser melting. Mater. Sci. Eng. A.

[64] Fu Z.H., Yang B.J., Shan M.L., Li T., Zhu Z.Y., Ma C.P., Zhang X., Gou G.Q., Wang Z.R., Gao W. (2020). Hydrogen embrittlement behavior of SUS301L-MT stainless steel laser-arc hybrid welded joint localized zones. Corros. Sci..

[65] Bhanu V., Gupta A., Pandey C. (2022). Role of A-TIG process in joining of martensitic and austenitic steels for ultra-supercritical power plants—A state of the art review. Nucl. Eng. Technol..

[66] Rogalski G., Świerczyńska A., Landowski M., Fydrych D. (2020). Mechanical and Microstructural Characterization of TIG Welded Dissimilar Joints between 304L Austenitic Stainless Steel and Incoloy 800HT Nickel Alloy. Metals.

[67] (1999). Welding—Non-Destructive Testing of Welded Joints—Visual Testing.

[68] (1999). Non-Destructive Testing—Penetrant Testing—General Principles.

[69] (2002). Non-Destructive Testing of Welded Joints—Ultrasonic Testing of Welded Joints.

[70] (2000). Welding—Destructive Testing of Metal Welded Joints—Macroscopic and Microscopic Testing of Welded Joints.

[71] (2018). Standard Practice for Presentation of Constant Amplitude Fatigue Test Results for Metallic Materials.

[72] (2015). Standard Practice for Conducting Force Controlled Constant Amplitude Axial Fatigue Tests of Metallic Materials.

[73] (2020). Metals—Tensile Test—Part 1: Test Method at Room Temperature.

[74] (2005). Welding—Welded Joints (Excluding Beam Welding) of Steel, Nickel, Titanium and Their Alloys-Quality Levels for Imperfections.

[75] (2010). Destructive Testing of Welds in Metallic Materials—Bending Test.

